# Association of Vitamin D Level and Maternal Gut Microbiome during Pregnancy: Findings from a Randomized Controlled Trial of Antenatal Vitamin D Supplementation

**DOI:** 10.3390/nu15092059

**Published:** 2023-04-25

**Authors:** Andrea Aparicio, Diane R. Gold, Scott T. Weiss, Augusto A. Litonjua, Kathleen Lee-Sarwar, Yang-Yu Liu

**Affiliations:** 1 Channing Division of Network Medicine, Department of Medicine, Brigham and Women’s Hospital and Harvard Medical School, Boston, MA 02115, USA; andrea.aparicio@channing.harvard.edu (A.A.); drgold@bwh.harvard.edu (D.R.G.); scott.weiss@channing.harvard.edu (S.T.W.); 2 Department of Environmental Health, Harvard T.H. Chan School of Public Health, Boston, MA 02115, USA; 3 Division of Pediatric Pulmonary Medicine, Golisano Children’s Hospital at University of Rochester Medical Center, Rochester, NY 14642, USA; augusto_litonjua@urmc.rochester.edu; 4 Division of Allergy and Clinical Immunology, Brigham and Women’s Hospital and Harvard Medical School, Boston, MA 02115, USA; 5 Center for Artificial Intelligence and Modeling, The Carl R. Woese Institute for Genomic Biology, University of Illinois at Urbana-Champaign, Champaign, IL 61801, USA

**Keywords:** vitamin D supplementation, pregnancy, maternal gut microbiome

## Abstract

Shifts in the maternal gut microbiome and vitamin D deficiency during pregnancy have been associated, separately, with health problems for both the mother and the child. Yet, they have rarely been studied simultaneously. Here, we analyzed the gut microbiome (from stool samples obtained in late pregnancy) and vitamin D level (from blood samples obtained both in early and late pregnancy) data of pregnant women in the Vitamin D Antenatal Asthma Reduction Trial (VDAART), a randomized controlled trial of vitamin D supplementation during pregnancy, to investigate the association of vitamin D status on the pregnant women’s microbiome. To find associations, we ran linear regressions on alpha diversity measures, PERMANOVA tests on beta diversity distances, and used the ANCOM-BC and Maaslin2 algorithms to find differentially abundant taxa. Analyses were deemed significant using a cut-off *p*-value of 0.05. We found that gut microbiome composition is associated with the vitamin D level in early pregnancy (baseline), the maternal gut microbiome does not show a shift in response to vitamin D supplementation during pregnancy, and that the genus *Desulfovibrio* is enriched in women without a substantial increase in vitamin D level between the first and the third trimesters of pregnancy. We conclude that increasing the vitamin D level during pregnancy could be protective against the growth of sulfate-reducing bacteria such as *Desulfovibrio*, which has been associated with chronic intestinal inflammatory disorders. More in-depth investigations are needed to confirm this hypothesis.

## 1. Introduction

Vitamin D has been strongly associated with many conditions of human health. Initially, it was discovered to promote bone health [1,2], but now, it is known to have many other physiological functions, among which, importantly, are modulating immunological and inflammation responses [3,4,5]. Vitamin D can be obtained from supplements, diet, or sun exposure and is converted by the liver into its serum form 25-hydroxyvitamin D, which, in turn, is converted into its activated form 1,25-dihydroxyvitamin D [6] by the kidneys and many other cells in the body. The biological activity of vitamin D’s active form in the body is mediated by the vitamin D receptor (VDR), primarily expressed in the intestines, kidneys, parathyroid gland, and bones, though also expressed by several cells of the immune system [7]. In the gut, a growing amount of evidence indicates that vitamin D and VDR signaling are responsible for maintaining epithelial integrity and modulating immune and inflammatory responses when the barrier is penetrated by bacteria, leading to chronic states of inflammation when the signaling is disrupted [6,8].

The gut microbiome is composed of trillions of microbes that collectively influence the health of its host [9]. In particular, the gut microbiome is known to have important immunoregulation functions [10], and disruptions of it are associated with chronic inflammatory diseases [11,12]. Strong evidence that vitamin D is associated with microbiota alterations exists, but the results are considerably heterogeneous [13,14,15].

The gut microbiome composition changes with age and is naturally modified during several stages of life. During pregnancy, the microbiome shift often resembles diseased states or dysbiosis that, surprisingly, instead of leading to a decline in fitness, promotes homeostasis [16]. The gut microbiome is dramatically modified between the first and third trimesters of pregnancy, and bacteria related to inflammation and immune response processes are usually elevated as the pregnancy progresses [17,18]. 

The impact on the offspring of the maternal vitamin D level in serum and of vitamin D consumption during pregnancy has been studied several times, establishing associations with the children’s microbiome and with outcomes such as changes in the immune system modulation, asthma/allergic disease incidence, likelihood of *C. difficile* colonization, and changes in microbiome richness and composition [19,20,21]. However, to our knowledge, no clinical trials have focused on the impact of vitamin D consumption on the mothers’ own microbiome during pregnancy. One observational study found an association between vitamin D dietary intake and a reduction of microbiome richness and an increase of bacteria with proinflammatory properties [22]; however, due to the lack of serum vitamin D level measurements, and considering that its absorption may depend on many factors other than only diet, the knowledge gap persists. Changes in the maternal gut microbiome are known to be associated with the development of preeclampsia, gestational diabetes, and other serious pregnancy pathologies [23] and with negative offspring outcomes, especially with those related to immunity or allergic diseases [24,25,26]. Thus, exploring the effects of the maternal vitamin D status on their microbiome is not only useful in identifying potential associated pregnancy complications or impacts on the mother’s health. Additionally, it has been repeatedly shown that the maternal and infant microbiome are related [24,25,27,28], and therefore, this direction of research can also contribute to discovering potential consequences for the children’s health. 

Here, we analyze the 16S rRNA data generated from stool samples of 114 pregnant women in the Vitamin D Antenatal Asthma Reduction Trial (VDAART), a multi-site randomized, double-blind, placebo-controlled trial of vitamin D supplementation during pregnancy, to look for associations between the participants’ vitamin D status and their gut microbiome during the third trimester of pregnancy. We use three different measures for the vitamin D status: (1) baseline vitamin D level, (2) treatment assignment, and (3) change in vitamin D level over the trial period. In the following, we present three key findings. First, the baseline vitamin D level is associated with the gut microbiome composition. Second, the gut microbiome is robust enough such that the vitamin D supplementation (even with up to 4400 IU cholecalciferol daily) during pregnancy does not significantly modify it. Third, *Desulfovibrio,* a genus of Gram-negative sulfate-reducing bacteria, is enriched in women whose vitamin D level did not have a substantial increase (less than 10 ng/mL) over the trial period.

## 2. Materials and Methods

### 2.1. Vitamin D Antenatal Asthma Reduction Trial (VDAART)

VDAART was a randomized controlled trial of Vitamin D supplementation during pregnancy to prevent asthma in offspring conducted in the United States (St. Louis, Boston, and San Diego; NCT00920621). Eight hundred and seventy women were enrolled at 10–18 weeks of gestation and randomized to receive either a high dose of vitamin D oral supplements (4400 IU cholecalciferol daily, called the treatment or high-dose treatment hereafter) or a placebo dose of vitamin D oral supplements (400 IU cholecalciferol daily) between enrollment and delivery. The study protocol was approved by the institutional review boards at each participating institution and at Brigham and Women’s Hospital. All participants provided written informed consent [29].

### 2.2. Stool Sample Collection and Processing 

During the third trimester of pregnancy (weeks 32–38 gestation), 120 participants of the VDAART trial provided a stool sample. Subjects were asked to collect a 0.5 teaspoon-sized sample 1 to 2 days before a study visit and store the sample in a home freezer before transport with a freezer pack to the study site. Stool was not collected if participants had used antibiotics in the previous 7 days. After delivery to the study site, stool samples were immediately stored at −80 °C. Microbiome profiling was performed by sequencing the 16S rRNA hypervariable region 4 (V4 515F/816R region) on the Illumina MiSeq platform at Partners Personalized Medicine (Boston, MA, USA). Of the 120 stool samples collected, 2 with fewer than 1000 reads measured were excluded from the analysis, leaving 118 samples.

### 2.3. Blood Sample Collection and Processing

Maternal blood samples were collected at two time points: at enrollment at 10–18 weeks of gestation and at 32–38 weeks of gestation. The serum 25-hydroxyvitamin D 25(OH)D level (termed as the vitamin D level hereafter) was measured in both samples using the DiaSorin LIAISON method (a chemiluminescence assay) at the Channing Division of Network Medicine, Brigham and Women’s Hospital (Boston, MA, USA). All the 118 women who provided a stool sample in the third trimester of pregnancy provided a blood sample at the first time point (yielding a baseline vitamin D level), and 114 of them also provided one at the second time point (yielding a final vitamin D level). Therefore, we have blood samples to analyze the change in vitamin D level between the early and late pregnancies of 114 women; 59 of them were in the treatment group, and the remaining 55 were in the placebo group. 

### 2.4. Serum Vitamin D Level

**Figure 1:**
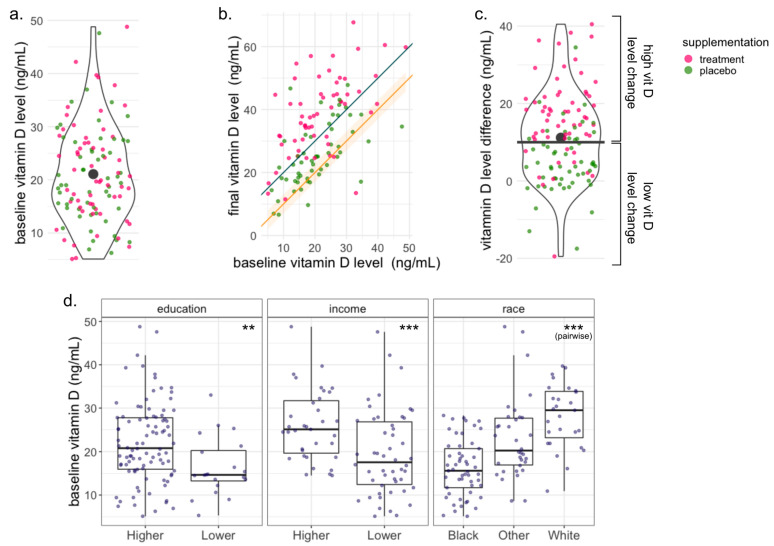
Vitamin D status was measured in three different ways: baseline vitamin D level (serum 25-hydroxyvitamin D 25(OH)D (ng/mL) measured in a blood sample at enrollment), treatment assignment (treatment or placebo vitamin D supplementation), and vitamin D level change over the trial period (high change or an increase of at least 10 ng/mL between the enrollment and third trimester and low change or a difference of less than 10 ng/mL). (**a**–**c**) The colors of the markers in the three panels represent the treatment assignment of every participant. (**a**) Distribution of the baseline vitamin D level of all the participants. The black marker represents the mean baseline vitamin D level for all participants (21.01 ng/mL). The treatment assignment is not associated with the baseline vitamin D level. (**b**) Baseline vitamin D level vs. final vitamin D level. The orange line and band trace a difference of 0 ± 3 ng/mL between the baseline and the final measurements. The gray line traces a difference of 10 ng/mL between baseline and final measurements. Most of the participants had an increase in their vitamin D level over the trial period. (**c**) Distribution of the Vitamin D level difference over the trial period. The black marker represents the mean vitamin D difference in all participants (11.27 ng/mL); the black horizontal line represents the cut-off of 10 ng/mL that separates the high and low change groups. (**d**) Distribution of the participants’ baseline vitamin D level stratified by the different characteristics. The baseline vitamin D value is significantly associated with the participants’ education, income, and race. Significance: ** *p*-value ≤ 0.01, *** *p*-value ≤ 0.00.

For the 114 women with baseline and final measurements (Figure 1a,b), we calculated the vitamin D level difference as the final level value minus the baseline level value; the mean vitamin D level difference among the 114 women was 11.2 ng/mL (Figure 1c). We used a rounded cut-off of 10 ng/mL to categorize the women into two groups: high change (difference of more than 10 ng/mL in serum 25-hydroxyvitamin D 25(OH)D) and low change (difference equal to or less than 10 ng/mL in serum 25-hydroxyvitamin D); sixty-two women fell into the high change group and fifty-two in the low change group. Additionally, we created a variable that simultaneously accounts for the change in vitamin D level and the baseline vitamin D level (see the SI for the details and corresponding analysis). 

### 2.5. Mothers’ Characteristics

The stool and blood samples were collected at three different sites: Boston, MA; San Diego, CA; and St. Louis, MO. The available subjects’ characteristics from the initial enrollment questionnaire are the mothers’ age, race, education, household income, and history of asthma and hay fever. Mothers were categorized into groups for each factor, except for their age, which was kept as a continuous variable. Race and ethnicity information was collected, because they are determinants of the circulating 25-hydroxyvitamin D levels; the race and ethnicity of every participant was self-reported. Participants were asked to first categorize themselves as either Hispanic or non-Hispanic, then to categorize their race into prespecified categories. Race/ethnicity (called race hereafter) groups were collapsed into 3 groups for the analysis: Black or African American mothers (called Black mothers hereafter), White, non-Hispanic mothers (called White mothers hereafter), and Hispanic mothers and mothers of other races (*n* = 25 and *n* = 9, respectively, called Other race hereafter). Education groups: higher education (some college education or more, *n* = 94) and lower education (technical school, high-school, or less, *n* = 20). Income groups: higher income (household income of more than 50,000 USD/year, *n* = 35) and lower income (less than 50,000 USD/year, *n* = 50). History of asthma or hay fever: yes (*n* = 39, *n* = 65, resp.) or no (*n* = 65, *n* = 49, resp.). 

### 2.6. Statistical Analyses

For the statistical analyses, we measured the vitamin D status in three different ways: baseline vitamin D level (measured at enrollment), randomized treatment assignment (treatment or placebo supplementation), and vitamin D level change between the baseline and final measurements (high or low change). Unless specifically noted, we used a cut-off *p*-value of 0.05 to deem the results statistically significant for all our analyses. All analyses of microbiome associations were adjusted for two a priori selected potential covariates: participants’ race and education level, unless otherwise specified.

#### 2.6.1. Alpha Diversity

Alpha diversity measures are estimates of an individual sample’s taxonomic diversity. We computed the observed richness, Shannon, and Simpson indices using the Phyloseq package in R [30]. The observed richness simply counts the number of different taxa present in each sample. The Shannon and Simpson indices incorporate the measures of richness and evenness of every sample. We used Wilcoxon rank-sum tests and covariate adjusted linear regressions to compare the samples’ estimates of these three alpha diversity metrics by measures of the vitamin D status.

#### 2.6.2. Beta Diversity

Beta diversity measures quantify the dissimilarity in taxonomic composition between two samples. We computed the following measures: Bray–Curtis dissimilarity, Jaccard distance, Unifrac distance, and Weighted-Unifrac distance using the Phyloseq package in R. We used the adonis2 algorithm in the R package vegan [31], which performs PERMANOVA tests using the distance matrices to find associations between the beta diversity dissimilarity measurements and measures of the maternal vitamin D status.

#### 2.6.3. Abundance Association Analysis

To ensure robustness of the identified associations, we used two different methods and their corresponding R packages, which look for associations between the subject variables and the abundance of specific taxa: ANCOM-BC [32] and MaAsLin [33]. ANCOM-BC is a differential abundance method that uses a log-linear regression framework with a sample-specific offset term to account for differences in the sampling fraction between samples. MaAsLin is a multivariate association method that uses additive linear models to detect associations between specific groups and the abundance of taxa, simultaneously treating all the present taxa as outcomes. We used the false discovery rate (FDR) method to adjust the *p*-values for multiple comparisons. The association analyses were performed on genus-level data.

## 3. Results

### 3.1. Subject Characteristics

The mean baseline vitamin D level in early pregnancy among all 114 participants was 21.08 ng/mL (Figure 1a). Table 1 summarizes the participants’ characteristics with respect to their baseline vitamin D level. We found a significant association between the baseline vitamin D level and the mothers’ race, income, and education level (ANOVA *p*-value *=* $3.6\times 10^{-9}$, $2.8\times 10^{-4}$, 0.01, respectively). Black women tended to have the lowest baseline vitamin D level, White women tended to have the highest, and Latino/Hispanics and women of other races tended to be in between; women with lower income and women with lower education tended to have lower levels of baseline vitamin D (Figure 1d). The race and education variables were included as covariates in all downstream analyses, while the household income was excluded from the covariates due to high missingness (29 women, i.e., 25% of our cohort did not provide income data) and because it is strongly correlated with maternal education and race (chi-square test *p*-values < $7\times 10^{-3}$ and $5\times 10^{-4}$, respectively). Additionally, we found a significant association between the collection site and the women’s baseline vitamin D level (ANOVA *p*-value < 0.001). The collection site is correlated with the subjects’ race (chi-square test *p*-value < 0.0005), and thus, it was also excluded from the covariates. The baseline vitamin D level was treated as a continuous variable in all the downstream analyses, except for the differential abundance algorithm ANCOM-BC, for which we used the mean baseline vitamin D level of 21.01 ng/mL as a cut-off to define two groups: below the mean and above the mean.

The participants of VDAART were randomized to receive either a high (4400 IU daily cholecalciferol) or a low (400 IU daily cholecalciferol) dose of vitamin D supplements (called treatment or placebo, respectively, hereafter) [34,35]. 

Table 2 summarizes the participants’ characteristics stratified by their treatment assignment, revealing that the randomization performed for the original VDAART cohort (>800 women) resulted in similar baseline characteristics between treatment groups among the 114 women with baseline and final vitamin D measurements. 

Most of the 114 women had an increased vitamin D level between early and late pregnancy, though the extent of the vitamin D increase varied between participants (Figure 1a). The mean difference between the baseline and final vitamin D level among the participants was 11.2 ng/mL. A cut-off of 10 ng/mL in the vitamin D level difference yielded two groups: high vitamin D level change (increase of at least 10 ng/mL) with 62 subjects and low vitamin D level change (decrease in vitamin D or increase of less than 10 ng/mL) with 52 subjects. We did not find any significant associations between the change groups and the participants’ characteristics, suggesting that the vitamin D level change was likely not confounded by them (Table S2). The vitamin D level in women in the treatment group (*n* = 59) increased by 16.8 ng/mL on average (48 of them fell in the high change group and 11 of them in the low change group), while the vitamin D level in the women in the placebo group (*n* = 55) increased by only 5.11 ng/mL on average (14 of them fell into the high change group and 41 of them in the low change group). The treatment assignment is significantly associated with the vitamin D level change assignment (chi-square test *p*-value $<6.7\times 10^{-9}$, Figure S3b). A significant vitamin D level difference between the treatment groups and the absence of an association with the baseline vitamin D level were also reported for the entire VDAART cohort [34]. We found that the vitamin D level change was not associated with the baseline vitamin D level (Figure S3a). To further reduce the risk of confounding, we ran all the downstream analyses for a variable baseline-change combination variable, with no qualitative changes to the results (SI). 

### 3.2. Microbiome Composition Is Associated with Baseline Vitamin D Level

**Figure 2:**
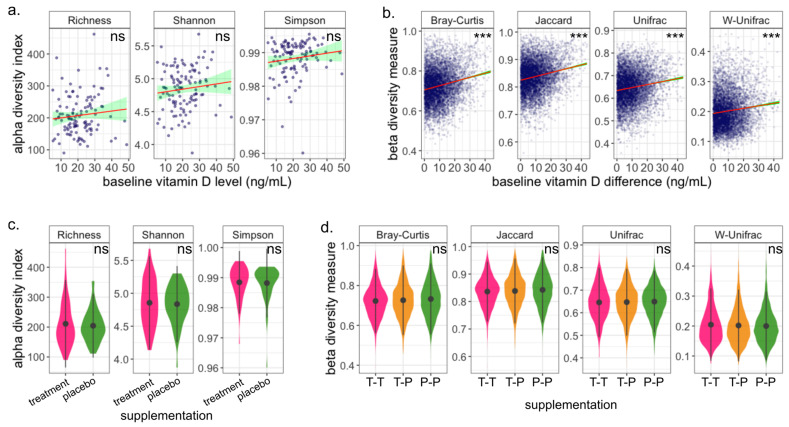
The baseline vitamin D is associated with the microbiome composition but not with its richness; the microbiome is robust for vitamin D supplementation. (**a**,**b**) The red line traces a linear model fit of the panel’s data, and the green band represents a 95% confidence interval. (**a**) The markers represent every individual’s baseline vitamin D level with respect to their alpha diversity. No significant association between the baseline vitamin D level and the alpha diversity indices was found. (**b**) Every marker represents the difference between the baseline vitamin D levels of two participants with respect to their corresponding beta diversity measurement. We found a significant association between the baseline vitamin D level and all the beta diversity indices that we analyzed (Bray–Curtis, Jaccard, Unifrac, and Weighted-Unifrac). Pairs of participants with a larger difference in baseline vitamin D levels tend to have more dissimilar microbiome compositions than pairs whose baseline vitamin D levels are closer to each other. (**c**,**d**) The black markers represent the mean values, and the black vertical lines represent the standard deviation. (**c**). Distribution of the alpha diversity indices among the two treatment groups. We found no association between the microbiome richness and the subjects’ treatment assignment for any of the analyzed alpha diversity indices analyzed (Observed, Shannon, and Simpson diversity). (**d**) The magenta (green) violins represent the distribution of inter-individual gut microbiome dissimilarity of subjects assigned to the high dose of vitamin D supplementation group (placebo group), labeled as T-T (P-P). The yellow violins represent the distribution of gut microbiome dissimilarities between subjects assigned to the treatment group and those assigned to the placebo group, labeled as T-P. There is no association between the treatment assignment and any of the dissimilarity measures. Significance: *** *p*-value ≤ 0.001.

We found a small but not statistically significant positive correlation between the baseline vitamin D level and gut microbiome richness or for the three alpha diversity indices analyzed (Figure 2a). We found a significant association between the women’s baseline vitamin D level and their microbiome composition for all beta diversity measurements (Bray–Curtis, Jaccard, Unifrac, and W-Unifrac adonis2 (PERMANOVA); *p*-values < 0.025, 0.025, 0.015, and 0.045, respectively). This association was not modified in models adjusted for education and race (Bray–Curtis, Jaccard, Unifrac, and W-Unifrac-adjusted adonis2 (PERMANOVA); *p*-values < 0.015, 0.015, 0.015, and 0.035, respectively). A covariate-adjusted (education and race) linear regression between every pair of subjects’ differences in baseline vitamin D and the corresponding beta diversity distance between them confirmed the significant association (*p*-value < $2\times 10^{-16}$ for all distances, Figure 2b). However, specific individual taxa associated with baseline vitamin D were not identified by either the ANCOM-BC or MaAsLin2 algorithms adjusted for both education and race (all FDR > 0.05 at the genus level).

### 3.3. Gut Microbiome Is Robust to Vitamin D Supplementation during Pregnancy

In an intention-to-treat analysis, we found no significant associations between the participants’ treatment assignment and any of the alpha diversity indices that we calculated (Figure 2c), suggesting that the vitamin D supplementation did not modify the participants’ microbiome diversity during the trial period. We also did not find any significant associations with the analyzed beta diversity measurements (Figure 2d), suggesting that the microbiome composition was not altered with the short-term treatment during pregnancy. Additionally, both differential abundance algorithms, ANCOM-BC and MaAsLin2, failed to identify any taxa that was significantly differentially expressed between the groups (all FDR > 0.05). This suggests that the microbiome during pregnancy is robust for short-term vitamin D supplementation over the treatment period.

### 3.4. Change in Vitamin D Level Does Not Impact Microbiome Diversity

Comparing participants by the change in their vitamin D level allows for an assessment of the impact of vitamin D over a relatively short period (between weeks 10–18 and weeks 32–38 of pregnancy) on the microbiome and accounts for potential lack of adherence to the vitamin D supplementation treatment assignment. All the vitamin D level change analyses were adjusted for the participants’ baseline vitamin D level, in addition to their race and education. We found no significant associations between the participants’ vitamin D change and their microbiome richness for any of the alpha diversity indices that we analyzed. We also found no significant associations with the women’s microbiome composition for any of the analyzed beta diversity measurements. This suggests that the microbiome richness and composition are robust to changes in the vitamin D level during pregnancy.

### 3.5. Desulfovibrio Is Enriched in Pregnant Women with Low Change of Vitamin D Level

**Figure 3:**
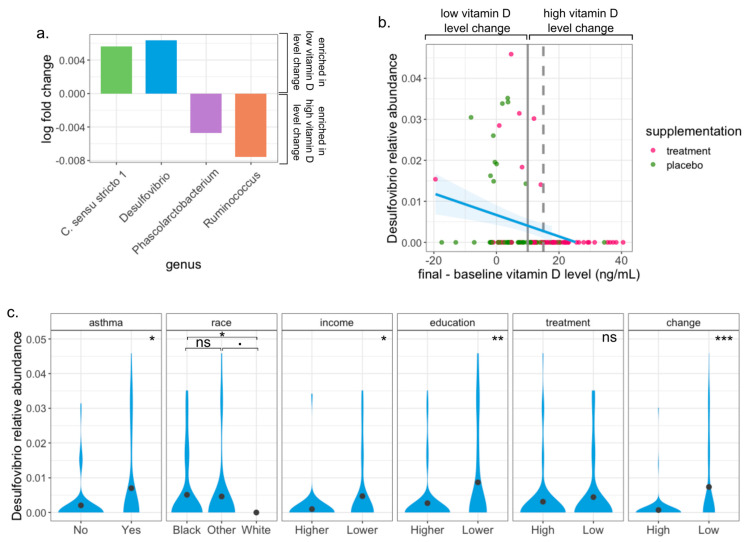
The sulfate−reducing bacteria *Desulfovibrio* is overexpressed in subjects with low vitamin D level change. (**a**) Log fold change of differentially abundant genera. Two genera are enriched in the low change group, and two are depleted (significant ANCOM-BC *p*-value). When adjusting for multiple comparisons, the association remains significant only for the overexpression of *Desulfovibrio* (significant ANCOM-BC q-value). (**b**) Vitamin D level change (final - baseline measurement) vs. *Desulfovibrio* relative abundance. The vertical solid line represents the 10 ng/mL cut-off that defines the high and low change groups. *Desulfovibrio* is almost exclusively present in participants with low vitamin D level change (left of solid vertical line) and is absent in those that had a vitamin D level difference larger than 15 ng/mL (discontinuous vertical line). The blue line traces a lineal model regression fit with a 95% confidence interval (blue transparency). (**c**) *Desulfovibrio* relative abundance distribution with respect to participants’ characteristics. A significant association exists between *Desulfovibrio*’s abundance and the subjects’ history of asthma, race, income, education, and vitamin D level change. No significant association with the subject’s treatment assignment was found. *Desulfovibrio* was not present in White women. Significance: ^**∙**^ *p*-value ≤ 0.1, * *p*-value ≤ 0.05, ** *p*-value ≤ 0.01, *** *p*-value ≤ 0.001.

Next, we looked at differentially abundant taxa between the change groups at the genus level. We found that two genera were enriched in the low change group (*Clostridium* sensu *stricto 1* and *Desulfovibrio*, ANCOM-BC *p*-values < 0.04 and 0, respectively) and that two genera were depleted (*Coprococcus* and *Fusicatenibacter*, ANCOM-BC *p*-value < 0.02 for both) (Figure 3a). However, after adjusting for multiple comparisons, only *Desulfovibrio* remained significantly differentially abundant (ANCOM-BC FDR < 0.01, log fold change = 0.0064). The MaAsLin analysis also revealed an enrichment in *Desulfovibrio* in women with low change in vitamin D (coefficient of 0.04 and *p*-value < $8\times 10^{-5}$, q-value < 0.01, blue line in Figure 3b). Indeed, *Desulfovibrio* was expressed almost exclusively in participants that had a low change in their vitamin D level (area on the left of solid gray line in Figure 3b), and it was not expressed in any of the participants whose vitamin D level increased by 15 ng/mL or more (area on the right of the discontinuous line in Figure 3b). Finally, we found significant associations between the relative abundance of *Desulfovibrio* and the participants’ history of asthma (Figure 3c, linear model adjusted for the education level coefficient of 0.0045 and *p*-value < 0.016), education, and income (Wilcoxon rank-sum test, *p*-value < 0.03 for both). We found a weak association between the abundance of *Desulfovibrio* and the participants’ race (ANOVA *p*-value = 0.671) and no significant association with the treatment assignment. In particular, *Desulfovibrio* was not present in stool samples from any White participant and was less abundant in participants with higher income and education compared to those with lower income and education. However, the significant associations between the abundance of *Desulfovibrio* and vitamin D change were preserved after adjusting the ANCOM-BC and MaAsLin algorithms for race and education as covariates. Noteworthy, a linear model adjusted for education level showed that there is no significant association between *Desulfovibrio* and the mothers’ baseline vitamin D level, but analyses simultaneously accounting for the change in vitamin D level and the baseline vitamin D level pointed toward an enrichment of *Desulfovibrio* among participants with a low increase in vitamin D over pregnancy (see the SI for the detailed results).

Interestingly, we found that the presence of *Desulfovibrio* is negatively, yet weakly, correlated with a low consumption of fruits when adjusted for education level (maternal diet summary derived from the principal component analysis of an 18-item food frequency questionnaire data. The second principal component represents a diet low in citrus and other fruits. Adjusted linear regression *p*-value = 0.0826). Additionally, we found that *Desulfovibrio* was present in a very small proportion (1.6%, *n* = 4) of the offspring gut microbiomes in 244 children who provided a stool sample between 3 and 6 months of age. None of the infants with a presence of *Desulfovibrio* were born to White mothers, mothers with higher income, or had developed asthma at 3 or 6 years old. 

## 4. Discussion

Here, we described the vitamin D status in pregnant women in three different ways (baseline vitamin D level, randomization to high- or low-dose vitamin D supplementation, and vitamin D level change over the trial period), each one shedding light on the relationship with the gut microbiome in its own way. 

The baseline vitamin D level (measured at enrollment at 10–18 weeks of gestation) provides a perspective of the mothers’ background in terms of their vitamin D status. For example, it might reflect their eating habits, history of supplementation, exposure to the sun, and/or ability to process the acquired vitamin D through any of these mechanisms. The identified association between the participants’ baseline vitamin D level and their race and education level is in agreement with previous findings that indicate a higher risk of vitamin D deficiency in non-Whites [36,37] and in individuals with a lower education level [36]. Additionally, a lower income, which is strongly associated with a lower education level and non-White ethnicity in our data, is known to be associated with a lower vitamin D intake. We found a small and not statistically significant positive correlation between the baseline vitamin D level and the alpha diversity and a significant association between the baseline vitamin D level and the subjects’ microbiome composition. The latter has been associated with the development of allergies, asthma, or other respiratory diseases [38]. Since an adequate vitamin D level has been iteratively found to be protective against airway inflammation processes [39], immune-mediated disorders [4,40], and inflammation [5], our findings may help fill in some of the mechanistic gaps in the microbiome–vitamin D–asthma axis. 

VDAART was originally designed to evaluate the impact of prenatal maternal vitamin D supplementation on asthma and respiratory diseases in children [29,34,35], but it also provides an opportunity to evaluate other effects of the treatment assignment. We found that, while most mothers’ vitamin D levels increased over the trial period, especially so for those in the treatment group, their microbiome composition seemed to not be affected by the treatment assignment. This suggests that vitamin D supplementation during pregnancy might be beneficial for the mothers in protecting them against vitamin D deficiency-related disorders and, potentially, for their offspring [21,22,41] without impacting the mothers’ risk with respect to microbiome shifts during the pregnancy period. However, depending on the chronicity of vitamin D deficiency prior to pregnancy, there may be physiologic adaptations to the deficiency that may need longer periods of supplementation prior to pregnancy to effect a change in the mothers’ vitamin D levels. Consequently, and based on our findings of an association between the baseline vitamin D level and microbiome composition, in order for changes in the microbiome to be consistently detected across different microbiome-related downstream analyses, a longer supplementation period, in addition to longer periods between measurements, might be needed. 

The vitamin D level in people under a supplementation schedule not only depends on their assigned dose but can be determined by a multitude of factors, such as their vitamin D level at baseline, sunlight exposure, diet, the liver’s efficiency in converting the absorbed vitamin D into its serum form, adherence to the treatment, the amount of time their vitamin D level has been in a (in)sufficient range, and certain health conditions or genetic predispositions [42]. Therefore, the change in vitamin D level over a certain period allows a more accurate vision of its associations with the microbiome than information on the vitamin D intake via supplements or diet. We found enrichment of the genus *Desulfovibrio* in subjects whose vitamin D levels had low change over the trial period. *Desulfovibrio* is the most common sulfate-reducing bacteria in the human intestine [43] and is also found in water sediments and environments with extreme temperature or pH [44]. In humans, the presence of *Desulfovibrio* has been associated with an increased incidence of inflammatory bowel diseases [45,46] and may even have a causal role in their pathophysiology [47,48]. Our results might indicate that an adequate vitamin D level can be protective against a higher risk of developing IBD or other related conditions by way of modulating the abundance of this gut microbe. This result agrees with previous studies that found negative correlations between *Desulfovibrio’s* abundance and vitamin D dietary [49] and supplements [50] intake. Moreover, the inability to absorb and process vitamin D has been found to cause dysbiosis and the development of sulfate-induced colitis in mice [51]. However, this pathway requires further clarification; for example, our data contradicts the results of an observational study that found a positive correlation between vitamin D dietary intake and *Desulfovibrio* abundance [52]. Interestingly, some evidence of negative correlations between *Desulfovibrio* and respiratory ailments are available [53,54], but we found a positive correlation with the mothers’ history of asthma. On the other hand, we found that no children in the VDAART trial with asthma had a presence of *Desulfovibrio* in their gut microbiome, though the genus was found in only a tiny proportion of the subjects. Thus, more studies in this direction are necessary to establish a conclusive association. Our data revealed an increased prevalence of *Desulfovibrio* in participants of more disadvantaged backgrounds (lower income and education level), as well as an important difference between the race groups. To our knowledge, this link has not been explored in depth before, and studies that aim to address this gap would help establish an association. 

Some limitations of this study are the relatively small sample size; short trial duration; and the lack of metagenomic sequencing, stool microbiome data from the first trimester of pregnancy, and detailed diet information. The small sample size could potentially decrease the generalizability of our results, although we do have a diverse cohort from geographically distant study sites. The duration of the study, both in terms of the supplementation duration and the time between baseline measurements and follow up, might hinder from detecting changes in the microbiome more robustly. 16s rRNA sequencing offers many advantages over shotgun metagenomic sequencing, such as the lower cost, sensitivity to contamination, and bioinformatic requirements, with the drawback that is limited to the 16s region of the rRNA gene only, leading to a loss in resolution in terms of taxonomic detection capabilities. While 16s rRNA sequencing has been the most prevalent genetic marker for decades, full gene sequencing data would allow a more in-depth analysis of the role that vitamin D plays in the microbiome during pregnancy. The availability of gut microbiome data at enrollment would provide a more accurate comparison of the vitamin D supplementation effects on the microbiome by establishing a baseline microbiome status for every participant. Finally, the dietary intake of vitamin D can play a role in the mothers’ vitamin D level. Therefore, more accurate information about the maternal diet could help distinguish better the effect of the supplementation. Detailed dietary information could also be leveraged to investigate the interaction between vitamin D and other compounds known to have an interplay with the gut microbiome and chronic diseases, such as polyphenols [55,56]. An additional limitation of our analysis is the exclusion of the mothers’ BMI, as the vitamin D levels have been found to be associated with changes in the BMI [57].

In summary, here. we have shown evidence that supports three main conclusions. First, our data suggest that that the vitamin D level in the serum is linked to the gut microbiome composition in pregnant women but not to its richness. Second, that the gut microbiome seems to be robust to vitamin D supplementation during pregnancy. Third, an increase in the vitamin D level between the first and third trimesters of pregnancy, in addition to all its other well-known health benefits, could protect against the growth of sulfate-reducing bacteria, such as *Desulfovibrio*, which is associated with several respiratory and bowel inflammation-related ailments. Our results suggest that, in order to observe a stronger effect of vitamin D supplementation on the gut microbiome of pregnant women, a longer supplementation period might be necessary, starting before the pregnancy occurs, and its length could depend on the chronicity of deficiency.

## Figures and Tables

**Table 1:** Summary of the participants’ characteristics with respect to their measurement of the serum 25−hydroxyvitamin D 25(OH)D level at enrollment (baseline vitamin D level).

			Baseline Vitamin D Level		
		Size (%)	Mean	SD	*p* Value
		114 (100)	21.08	9.07	
**Race**	Black	53 (46.5)	16.21	6.49	3.6 × 10^−9^
	Other	34 (29.8)	23.03	9.47	
	White	27 (23.7)	28.18	7.33	
**Household** **income**	50k or more	35 (30.7)	26.01	8.11	2.8 ×10 ^−4^
	Less than 50k	50 (43.9)	19.45	9.73	
	NA	29 (25.4)	17.93	6.41	
**Education**	College or more	94 (82.5)	22.08	9.21	0.010
	Highschool, technical school or less	20 (17.5)	16.36	6.77	
**Site name**	Boston	51 (44.7)	21.01	8.57	0.001
	San Diego	15 (13.2)	28.74	8.61	
	Saint Louis	48 (42.1)	18.76	8.56	
**Maternal** **asthma**	No	75 (65.8)	21.24	9.25	0.796
	Yes	39 (34.2)	20.77	8.82	
**Maternal** **hay fever**	No	49 (43.0)	21.04	10.01	0.971
	Yes	65 (57.0)	21.11	8.37	
**Maternal age**		27.56 (5.85)	27.56	5.85	0.326
**Difference vitamin D level**		11.18 (11.30)	11.18	11.3	0.796
**Treatment**	High dose	59 (51.8)	21.64	9.61	0.929
	Low dose	55 (48.2)	20.48	8.49	

**Table 2:** Summary of the participants’ characteristics with respect to the treatment group that they were assigned.

			Treatment Assignment		
		Overall	Treatment	Placebo	*p* Value
		114	59	55	
**Race**	Black	53 (46.5)	26 (44.1)	27 (49.1)	0.614
	Other	34 (29.8)	20 (33.9)	14 (25.5)	
	White	27 (23.7)	13 (22.0)	14 (25.5)	
**Household** **income (%)**	50k or more	35 (30.7)	19 (32.2)	16 (29.1)	0.927
	Less than 50k	50 (43.9)	25 (42.4)	25 (45.5)	
	NA	29 (25.4)	15 (25.4)	14 (25.5)	
**Education (%)**	College or more	94 (82.5)	48 (81.4)	46 (83.6)	0.941
	Highschool, technical school or less	20 (17.5)	11 (18.6)	9 (16.4)	
**Baseline** **vitamin D level (mean (SD))**		21.08 (9.07)	21.64 (9.61)	20.48 (8.49)	0.496
**Site name** **(%)**	Boston	51 (44.7)	29 (49.2)	22 (40.0)	0.543
	San Diego	15 (13.2)	8 (13.6)	7 (12.7)	
	Saint Louis	48 (42.1)	22 (37.3)	26 (47.3)	
**Maternal asthma (%)**	No	75 (65.8)	39 (66.1)	36 (65.5)	1.000
	Yes	39 (34.2)	20 (33.9)	19 (34.5)	
**Maternal hay fever (%)**	No	49 (43.0)	26 (44.1)	23 (41.8)	0.958
	Yes	65 (57.0)	33 (55.9)	32 (58.2)	
**Maternal age (mean (SD))**		27.56 (5.85)	27.47 (5.55)	27.65 (6.19)	0.871

## Data Availability

Microbiome sequencing data from VDAART are part of the ECHO consortium, and ECHO consortium members can obtain the data directly from the ECHO DCC or for those not part of ECHO directly from the authors. All other relevant data are available from the authors upon reasonable requests.

## Supplementary Materials

The following supporting information can be downloaded at: https://www.mdpi.com/article/10.3390/nu15092059/s1.

## References

[1] Holick M.F. (1996). Vitamin D and Bone Health. J. Nutr..

[2] Laird E., Ward M., McSorley E., Strain J.J., Wallace J. (2010). Vitamin D and Bone Health; Potential Mechanisms. Nutrients.

[3] DeLuca H.F. (2004). Overview of General Physiologic Features and Functions of Vitamin D. Am. J. Clin. Nutr..

[4] Charoenngam N., Holick M.F. (2020). Immunologic Effects of Vitamin D on Human Health and Disease. Nutrients.

[5] Guillot X., Semerano L., Saidenberg-Kermanac’h N., Falgarone G., Boissier M.-C. (2010). Vitamin D and Inflammation. Jt. Bone Spine.

[6] Fakhoury H.M.A., Kvietys P.R., AlKattan W., Anouti F.A., Elahi M.A., Karras S.N., Grant W.B. (2020). Vitamin D and Intestinal Homeostasis: Barrier, Microbiota, and Immune Modulation. J. Steroid Biochem. Mol. Biol..

[7] Wang Y., Zhu J., DeLuca H.F. (2012). Where Is the Vitamin D Receptor?. Arch. Biochem. Biophys..

[8] Ardesia M., Ferlazzo G., Fries W. (2015). Vitamin D and Inflammatory Bowel Disease. Biomed. Res. Int..

[9] Ley R.E., Peterson D.A., Gordon J.I. (2006). Ecological and Evolutionary Forces Shaping Microbial Diversity in the Human Intestine. Cell.

[10] Boirivant M., Amendola A., Butera A. (2008). Intestinal Microflora and Immunoregulation. Mucosal Immunol..

[11] Ferreira C.M., Vieira A.T., Vinolo M.A.R., Oliveira F.A., Curi R., Martins F.d.S. (2014). The Central Role of the Gut Microbiota in Chronic Inflammatory Diseases. J. Immunol. Res..

[12] Kennedy P.J., Cryan J.F., Dinan T.G., Clarke G. (2014). Irritable Bowel Syndrome: A Microbiome-Gut-Brain Axis Disorder?. World J. Gastroenterol..

[13] Bellerba F., Muzio V., Gnagnarella P., Facciotti F., Chiocca S., Bossi P., Cortinovis D., Chiaradonna F., Serrano D., Raimondi S. (2021). The Association between Vitamin D and Gut Microbiota: A Systematic Review of Human Studies. Nutrients.

[14] Akimbekov N.S., Digel I., Sherelkhan D.K., Lutfor A.B., Razzaque M.S. (2020). Vitamin D and the Host-Gut Microbiome: A Brief Overview. Acta Histochem. Cytochem..

[15] Waterhouse M. (2019). Vitamin D and the Gut Microbiome: A Systematic Review of in Vivo Studies. Eur. J. Nutr..

[16] Nuriel-Ohayon M., Neuman H., Koren O. (2016). Microbial Changes during Pregnancy, Birth, and Infancy. Front. Microbiol..

[17] Koren O., Goodrich J.K., Cullender T.C., Spor A., Laitinen K., Bäckhed H.K., Gonzalez A., Werner J.J., Angenent L.T., Knight R. (2012). Host Remodeling of the Gut Microbiome and Metabolic Changes during Pregnancy. Cell.

[18] Fuhler G.M. (2020). The Immune System and Microbiome in Pregnancy. Best Pract. Res. Clin. Gastroenterol..

[19] Sordillo J.E., Zhou Y., McGeachie M.J., Ziniti J., Lange N., Laranjo N., Savage J.R., Carey V., O’Connor G., Sandel M. (2017). Factors Influencing the Infant Gut Microbiome at Age 3–6 Months: Findings from the Ethnically Diverse Vitamin D Antenatal Asthma Reduction Trial (VDAART). J. Allergy Clin. Immunol..

[20] Drall K.M., Field C.J., Haqq A.M., de Souza R.J., Tun H.M., Morales-Lizcano N.P., Konya T.B., Guttman D.S., Azad M.B., Becker A.B. (2020). Vitamin D Supplementation in Pregnancy and Early Infancy in Relation to Gut Microbiota Composition and C. Difficile Colonization: Implications for Viral Respiratory Infections. Gut Microbes.

[21] Kassem Z., Sitarik A., Levin A.M., Lynch S.V., Havstad S., Fujimura K., Kozyrskyj A., Ownby D.R., Johnson C.C., Yong G.J. (2020). Maternal and Cord Blood Vitamin D Level and the Infant Gut Microbiota in a Birth Cohort Study. Matern. Health Neonatol. Perinatol..

[22] Mandal S., Godfrey K.M., McDonald D., Treuren W.V., Bjørnholt J.V., Midtvedt T., Moen B., Rudi K., Knight R., Brantsæter A.L. (2016). Fat and Vitamin Intakes during Pregnancy Have Stronger Relations with a Pro-Inflammatory Maternal Microbiota than Does Carbohydrate Intake. Microbiome.

[23] Gorczyca K., Obuchowska A., Kimber-Trojnar Ż., Wierzchowska-Opoka M., Leszczyńska-Gorzelak B. (2022). Changes in the Gut Microbiome and Pathologies in Pregnancy. Int. J. Environ. Res. Public Health.

[24] Kimura I., Miyamoto J., Ohue-Kitano R., Watanabe K., Yamada T., Onuki M., Aoki R., Isobe Y., Kashihara D., Inoue D. (2020). Maternal Gut Microbiota in Pregnancy Influences Offspring Metabolic Phenotype in Mice. Science.

[25] Vuillermin P.J., Macia L., Nanan R., Tang M.L., Collier F., Brix S. (2017). The Maternal Microbiome during Pregnancy and Allergic Disease in the Offspring. Semin. Immunopathol..

[26] Ishimwe J.A. (2021). Maternal Microbiome in Preeclampsia Pathophysiology and Implications on Offspring Health. Physiol. Rep..

[27] Lee-Sarwar K.A., Chen Y.C., Chen Y.Y., Kozyrskyj A.L., Mandhane P.J., Turvey S.E., Subbarao P., Bisgaard H., Stokholm J., Chawes B. (2023). The Maternal Prenatal and Offspring Early-Life Gut Microbiome of Childhood Asthma Phenotypes. Allergy.

[28] Dunlop A.L., Mulle J.G., Ferranti E.P., Edwards S., Dunn A.B., Corwin E.J. (2015). The Maternal Microbiome and Pregnancy Outcomes That Impact Infant Health: A Review. Adv. Neonatal. Care.

[29] Litonjua A.A., Lange N.E., Carey V.J., Brown S., Laranjo N., Harshfield B.J., O’Connor G.T., Sandel M., Strunk R.C., Bacharier L.B. (2014). The Vitamin D Antenatal Asthma Reduction Trial (VDAART): Rationale, Design, and Methods of a Randomized, Controlled Trial of Vitamin D Supplementation in Pregnancy for the Primary Prevention of Asthma and Allergies in Children. Contemp. Clin. Trials.

[30] McMurdie P.J., Holmes S. (2013). Phyloseq: An R Package for Reproducible Interactive Analysis and Graphics of Microbiome Census Data. PLoS ONE.

[31] Oksanen J., Simpson G., Blanchet F., Kindt R., Legendre P., Minchin P., O’hara R., Solymos P., Stevens M., Szoecs E. (2022). Vegan: Community Ecology Package, Package Version 2.6-4.

[32] Lin H., Peddada S.D. (2020). Analysis of Compositions of Microbiomes with Bias Correction. Nat. Commun..

[33] Mallick H., Rahnavard A., McIver L.J., Ma S., Zhang Y., Nguyen L.H., Tickle T.L., Weingart G., Ren B., Schwager E.H. (2021). Multivariable Association Discovery in Population-Scale Meta-Omics Studies. PLoS Comput. Biol..

[34] Litonjua A.A., Carey V.J., Laranjo N., Harshfield B.J., McElrath T.F., O’Connor G.T., Sandel M., Iverson R.E., Lee-Paritz A., Strunk R.C. (2016). Effect of Prenatal Supplementation With Vitamin D on Asthma or Recurrent Wheezing in Offspring by Age 3 Years: The VDAART Randomized Clinical Trial. JAMA.

[35] Litonjua A.A., Carey V.J., Laranjo N., Stubbs B.J., Mirzakhani H., O’Connor G.T., Sandel M., Beigelman A., Bacharier L.B., Zeiger R.S. (2020). Six-Year Follow-up of a Trial of Antenatal Vitamin D for Asthma Reduction. New Engl. J. Med..

[36] Zadshir A., Tareen N., Pan D., Norris K., Martins D. (2005). The Prevalence of Hypovitaminosis D Among US Adults: Data from the NHANES III. Ethn. Dis..

[37] Ginde A.A., Liu M.C., Camargo C.A. (2009). Demographic Differences and Trends of Vitamin D Insufficiency in the US Population, 1988–2004. Arch. Intern. Med..

[38] Peroni D.G., Nuzzi G., Trambusti I., Di Cicco M.E., Comberiati P. (2020). Microbiome Composition and Its Impact on the Development of Allergic Diseases. Front. Immunol..

[39] Ali N.S., Nanji K. (2017). A Review on the Role of Vitamin D in Asthma. Cureus.

[40] Baeke F., Takiishi T., Korf H., Gysemans C., Mathieu C. (2010). Vitamin D: Modulator of the Immune System. Curr. Opin. Pharmacol..

[41] Gale C.R., Robinson S.M., Harvey N.C., Javaid M.K., Jiang B., Martyn C.N., Godfrey K.M., Cooper C. (2008). Maternal Vitamin D Status during Pregnancy and Child Outcomes. Eur. J. Clin. Nutr..

[42] Tsiaras W., Weinstock M. (2011). Factors Influencing Vitamin D Status. Acta Derm. Venerol..

[43] Loubinoux J., Valente F.M., Pereira I.A., Costa A., Grimont P.A., Le Faou A.E. (2002). Reclassification of the Only Species of the Genus Desulfomonas, Desulfomonas Pigra, as Desulfovibrio Piger Comb. Nov. Int. J. Syst. Evol. Microbiol..

[44] Muyzer G., Stams A.J.M. (2008). The Ecology and Biotechnology of Sulphate-Reducing Bacteria. Nat. Rev. Microbiol..

[45] Kushkevych I., Dordević D., Kollar P., Vítězová M., Drago L. (2019). Hydrogen Sulfide as a Toxic Product in the Small–Large Intestine Axis and Its Role in IBD Development. J. Clin. Med..

[46] Loubinoux J., Bronowicki J.-P., Pereira I.A.C., Mougenel J.-L., Faou A.E. (2002). Sulfate-Reducing Bacteria in Human Feces and Their Association with Inflammatory Bowel Diseases. FEMS Microbiol. Ecol..

[47] Kushkevych I., Kos J., Kollar P., Kralova K., Jampilek J. (2018). Activity of Ring-Substituted 8-Hydroxyquinoline-2-Carboxanilides against Intestinal Sulfate-Reducing Bacteria Desulfovibrio Piger. Med. Chem. Res..

[48] Kushkevych I., Leščanová O., Dordević D., Jančíková S., Hošek J., Vítězová M., Buňková L., Drago L. (2019). The Sulfate-Reducing Microbial Communities and Meta-Analysis of Their Occurrence during Diseases of Small–Large Intestine Axis. J. Clin. Med..

[49] Weng Y.J., Gan H.Y., Li X., Huang Y., Li Z.C., Deng H.M., Chen S.Z., Zhou Y., Wang L.S., Han Y.P. (2019). Correlation of Diet, Microbiota and Metabolite Networks in Inflammatory Bowel Disease. J. Dig. Dis..

[50] Pham V.T., Fehlbaum S., Seifert N., Richard N., Bruins M.J., Sybesma W., Rehman A., Steinert R.E. (2021). Effects of Colon-Targeted Vitamins on the Composition and Metabolic Activity of the Human Gut Microbiome—A Pilot Study. Gut Microbes.

[51] Ooi J.H., Li Y., Rogers C.J., Cantorna M.T. (2013). Vitamin D Regulates the Gut Microbiome and Protects Mice from Dextran Sodium Sulfate–Induced Colitis. J. Nutr..

[52] Garg M., Hendy P., Ding J.N., Shaw S., Hold G., Hart A. (2018). The Effect of Vitamin D on Intestinal Inflammation and Faecal Microbiota in Patients with Ulcerative Colitis. J. Crohn’s Colitis.

[53] Bowerman K.L., Rehman S.F., Vaughan A., Lachner N., Budden K.F., Kim R.Y., Wood D.L., Gellatly S.L., Shukla S.D., Wood L.G. (2020). Disease-Associated Gut Microbiome and Metabolome Changes in Patients with Chronic Obstructive Pulmonary Disease. Nat. Commun..

[54] Sohn K.-H., Baek M., Choi S.-M., Bae B., Kim R.Y., Kim Y.-C., Kim H.-Y., Yi H., Kang H.-R. (2020). Alteration of Lung and Gut Microbiota in IL-13-Transgenic Mice Simulating Chronic Asthma. J. Microbiol. Biotechnol..

[55] Pandey K.B., Rizvi S.I. (2009). Plant Polyphenols as Dietary Antioxidants in Human Health and Disease. Oxid. Med. Cell Longev..

[56] Singh A.K., Cabral C., Kumar R., Ganguly R., Rana H.K., Gupta A., Lauro M.R., Carbone C., Reis F., Pandey A.K. (2019). Beneficial Effects of Dietary Polyphenols on Gut Microbiota and Strategies to Improve Delivery Efficiency. Nutrients.

[57] Perna S. (2019). Is Vitamin D Supplementation Useful for Weight Loss Programs? A Systematic Review and Meta-Analysis of Randomized Controlled Trials. Medicina.

