# Supplementary material for: Association of Vitamin D Level and Maternal Gut Microbiome during Pregnancy: Findings from a Randomized Controlled Trial of Antenatal Vitamin D Supplementation
Source: Nutrients. 2023 Apr 25;15(9):2059. doi: 10.3390/nu15092059 (PMC10181263; doi:10.3390/nu15092059)
Supplement: Supplementary file 1 [file nutrients-15-02059-s001.zip › nutrients-2366039-supplementary.pdf]

Association of Vitamin D Level and Maternal Gut Microbiome during Pregnancy: Findings from a Randomized Controlled Trial of Antenatal Vitamin D Supplementation

Andrea Aparicio [1], Diane R. Gold [1,2], Scott T. Weiss [1], Augusto A. Litonjua [3], Kathleen Lee-Sarwar [1,4,*] and Yang-Yu Liu [1,5,*]

[1] Channing Division of Network Medicine, Department of Medicine, Brigham and Women's Hospital and Harvard Medical School, Boston, MA 02115, USA; andrea.aparicio@channing.harvard.edu (A.A.); drgold@bwh.harvard.edu (D.R.G.); scott.weiss@channing.harvard.edu (S.T.W.)
[2] Department of Environmental Health, Harvard T.H. Chan School of Public Health, Boston, MA 02115, USA
[3] Division of Pediatric Pulmonary Medicine, Golisano Children's Hospital at University of Rochester Medical Center, Rochester, NY 14642, USA; augusto_litonjua@urmc.rochester.edu
[4] Division of Allergy and Clinical Immunology, Brigham and Women's Hospital and Harvard Medical School, Boston, MA 02115, USA
[5] Center for Artificial Intelligence and Modeling, The Carl R. Woese Institute for Genomic Biology, University of Illinois at Urbana-Champaign, Champaign, IL 61801, USA
* Correspondence: klee-sarwar@bwh.harvard.edu (K.L.-S.); yyl@channing.harvard.edu (Y.-Y.L.)

Supplemental Information

Fecal Bacterial Microbiome Profiling

Stool samples were collected from mothers during the third trimester of pregnancy. Subjects were asked to collect a 0.5 teaspoon-sized sample 1 to 2 days before a study visit and store the sample in a home freezer before transport with a freezer pack to the study site. Stool was not collected if participants had used antibiotics in the past 7 days. After delivery to the study site, stool samples were immediately stored at -80° C. Microbiome profiling was performed by sequencing the 16S rRNA hypervariable region 4 (V4 515F/816R region) on the Illumina MiSeq platform at Partners Personalized Medicine (Boston, MA). In detail, approximately 200 mg of stool per sample was resuspended in 1 mL InhibitEX Buffer from Qiagen QIAamp Fast DNA Stool Mini Kit (Qiagen, Catalogue # 51604) in tubes containing 0.1 mm silicon beads (Genesee Scientific, Catalogue # 31-212S1). Samples were disrupted using a Mini-Beadbeater 24 from Biospec Products for 3 minutes. Extraction continued on the contents of the entire tube using the Qiagen QIAamp Fast DNA Stool Mini Kit automated on a Qiagen QIAcube. Resulting DNA quality control was evaluated using a PicoGreen assay (Quant-iT dsDNA assay kit, Thermofisher, catalogue number P7589) on a Gemini XP spectrophotometer from Molecular Devices.

Input into library construction was 15 ng DNA using NEXTflex 16S V4 Amplicon-Seq kit 2.0 (Bioo Scientific, Catalogue # 4203-04). Finished libraries were normalized to 10 nM using PicoGreen quantitation and sizing information gathered using a Tapestation D100 screen tapes and reagents (Agilent, Catalogue # 5067-5582 and 5067-5583). Final quality control was carried out to establish the amount of library containing ligated Illumina adaptors using KAPA Library Quantification Kits (KAPA biosystems, Catalogue # KK4824). Up to 288 libraries were pooled to provide equimolar amounts of each library in the pool, which was then run on Illumina MiSeq using MiSeq Reagent Kit v3 600 cycle kit (MS-102-3003). Each pool contained a 20% spike-in of PhiX Control library v3 (Illumina, Catalogue # FC-110-3001) to increase diversity of the library sequence. FASTQ files were generated on the MiSeq instrument.

Primer and adapter trimming was performed using Skewer. Chimera checking and filtering were performed using Qiime2 [1]. Reads were denoised using DADA2 as implemented in Qiime2 [2]. Processing of microbiome data was performed using Qiime [3] and Phyloseq package for R [4]. Of 1,327 stool samples with 16S rRNA microbial profiling, the median read count was 11,068 (interquartile range 9,063-13,868).

Taxonomy was assigned to representative sequences using a naive Bayes classifier pre-built from the 99% SILVA 138 database specific to the 515F/816R region for bacterial data [5,6]. A total of 14,488 amplicon sequencing variants (ASVs) were detected, corresponding to 402 genera.

Out of 120 maternal samples available, 2 had less than 1000 reads and were discarded. ASVs present in offspring samples but not present in any maternal sample were excluded, yielding 4,170 ASVs. To reduce spurious associations with rare taxa, for abundance association analyses, we included only fecal microbial genera detected in at least 10% of samples with read counts of at least 3: yielding 415 ASVs corresponding to 67 genera (Diagram S1).

Measurement of 25(OH)D

Maternal vitamin D level (total 25-hydroxyvitamin D (25(OH)D)) was measured at two time points: at 10-18 weeks of pregnancy (early pregnancy), and at 32-38 weeks (late pregnancy), according to the VDAART protocol. Circulating 25(OH)D in all maternal samples was determined using the DiaSorin Liaison LIAISON (Saluggia, Italy) chemiluminescence immunoassay at the Channing Division of Network Medicine, Brigham and Women's Hospital [7].

Statistical analyses

All statistical analyses were performed using R version 4.2.1.

Alpha Diversity

Alpha diversity measures (Observed, Shannon, and Simpson) were calculated using the `estimate_richness` function in the phyloseq R package [4]. The fitted line and confidence interval in Figure 2.a was generated using a linear model of the pictured data, with the `stat_smooth` function in the ggplot R package.

Beta Diversity

Beta diversity measures were calculated using the distance function in the phyloseq R package [4]. The Jaccard distance is calculated as the difference between the sizes of the union and the intersection of the sample sets divided by the size of the union of the sample sets. The Bray-Curtis dissimilarity is calculated as the ratio between the number of common species and the sum of the total species in both samples. The Unifrac and Weighted-Unifrac distances are calculated as the ratio between the sum of unshared branch lengths on a phylogenic tree between two samples and the sum of all branch lengths. The unweighted distance accounts for present and absent taxa while the weighted one accounts for the observed taxa counts. The obtained beta diversity dissimilarity measurements were compared to the three measures of maternal vitamin D status in covariate-adjusted adonis PERMANOVA tests (*vegan* R package [4]). Additionally, we calculated the difference between the baseline vitamin D level of every pair of subjects i.e., for subjects labeled 1 and 2:

$$B1 = \text{baseline vitamin D level of subject 1}$$
$$B2 = \text{baseline vitamin D level of subject 2}$$
$$\text{baseline vitamin D difference} = abs(B1 - B2).$$

We compared the difference in baseline vitamin D level between all possible pairs of subjects to the computed beta diversity distances in a covariate adjusted linear model (Figure 2b). The fitted line and confidence interval in Figure 2.b was generated using a linear model of the pictured data, with the `stat_smooth` function in the ggplot R package.

Supplemental results

A baseline and change in vitamin D level combination variable accounts for potential confounding by baseline vitamin D level.

The Vitamin D level in humans may depend on many different factors such as intake from diet or supplements, sunlight exposure, the liver's efficiency in converting the absorbed vitamin D into its serum form, certain health conditions, or genetic predisposition. Further, the change in vitamin D level over a certain period may also be affected by the initial level and for how long that has been maintained in the past, i.e., amount of time in a sufficient or deficient range. Additionally, even when a significant change in vitamin D level is detected, the duration of pregnancy might not be sufficient to induce a significant microbiome shift. Therefore, and to account for potential confounding by baseline vitamin D level on our vitamin D level change results, we created a new categorical variable that combines the baseline vitamin D level and its change over the pregnancy (called *baseline-change* hereafter). We used the mean baseline vitamin D level in the 114 subjects (21 ng/mL) as a cut-off for the baseline vitamin D level, and a variation of 10ng/mL between the baseline and final vitamin D levels as a cut-off for the change which yielded four levels: *High baseline – High change* (n=29), *High baseline – Low change* (n=22), *Low baseline – High change* (n=33), *and Low baseline – Low change* (n=30). The baseline-change is significantly associated with treatment assignment (Chi squared test p-value $< 3 \times 10^{-8}$, Figure S1.a).

Microbiome composition is associated with the combination of baseline vitamin D level and its change after supplementation.

We found no significant association between the baseline-change and the and gut microbiome richness or for any of the three alpha diversity indices analyzed (Figure S1.b). We found a significant association between the women's baseline-change and their microbiome composition for the W-Unifrac distance adjusted for race and education (covariate adjusted adonis2-PERMANOVA- p-value < 0.03), and a weak association for the rest of the beta diversity measurements (covariate adjusted adonis2 - PERMANOVA- p-values < 0.08 for Bray-Curtis, Jaccard, and Unifrac distances, Figure S1.c-d). The dissimilarity measures between pairs of subjects that fall into the same group are considered *within group* comparisons (Figure S1.c). For example, the Bray-Curtis dissimilarity between all possible pairs of subjects that fall into the High baseline – High Change group, whose distribution is pictured by the pink violin plots in Figure S1.c. On the other hand, dissimilarity measures between pairs of subjects that fall into different groups are considered *between group* comparisons (Figure S1.d). For example, the Bray-Curtis dissimilarity between all possible pairwise combinations of subjects that fall into the High baseline – High change and those that fall into the High baseline – Low change groups, whose distribution is pictured by the lime-green violin plots in Figure S1.d. We found a significant difference between all the within group and all the between group comparisons for all the beta diversity measurements (ANOVA pvalue < 0.001 for for Bray-Curtis, Jaccard, Unifrac and W-Unifrac).

Desulfovibrio is enriched in women with low baseline and low change in vitamin D level

We found that the *Desulfovibrio* genus is significantly differentially abundant among the baseline – change groups (covariate adjusted Maaslin coefficient = 0.06, q-value < 0.05). In particular, the abundance of *Desulfovibrio* in the Low baseline – Low change is significantly higher than in the High baseline – High change and in the Low baseline – High change groups (Figure S2, pairwise Wilcoxon rank sum tests adjusted for multiple comparisons p-value < 0.005 for both).

Diagrams

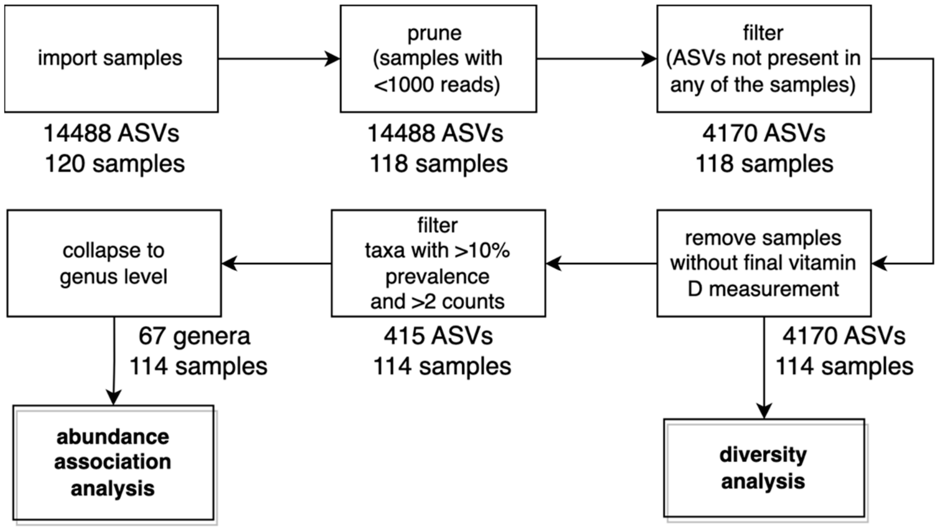

Diagram S1 Data pre-processing workflow.

Supplemental figures

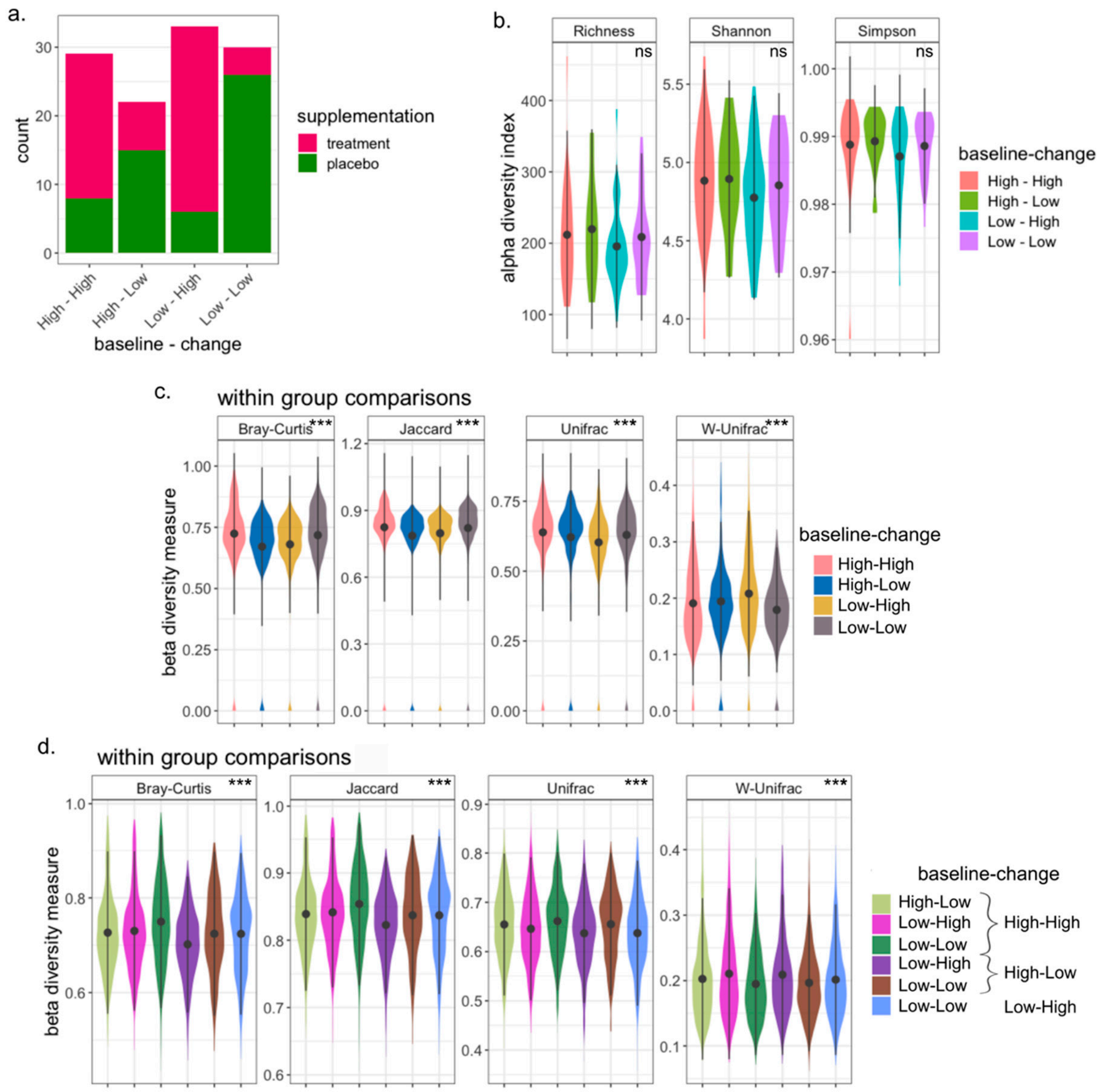

Figure S1: A variable that combines the baseline and the change in vitamin D level accounts for potential confounding by the baseline vitamin D level on the change in vitamin D level. Participants with a baseline vitamin D level above (below) the mean of 21.08 ng/mL were placed in a *High (Low) baseline* group. Participants with a change higher than 10 ng/mL were placed in a *High change* group, and those with a maximum change of 10 ng/mL were placed in a *Low change* group. In total there are four baseline – change groups: High (baseline) – High (change), High – Low, Low – High, and Low – Low. **a.** Baseline – change stratification of the 114 participants. The baseline – change variable is significantly associated with the treatment assignment (Chi squared test p-value $< 3 \times 10^{-8}$). **b.** No significant association between the baseline – change and the

microbiome richness for any of the alpha diversity indices. **c**. The microbiome composition is significantly associated with baseline-change in vitamin D level when separated into within and between group comparisons (ANOVA p-value < 6×10^{-6} for all beta diversity measurements).

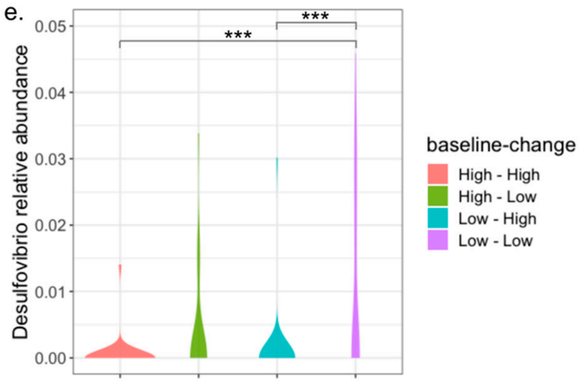

Figure S2 The Desulfovibrio genus is significantly differentially abundant among the baseline – change groups (covariate adjusted Maaslin q-value < 0.05). The abundance of *Desulfovibrio* in the Low baseline – Low change group is significantly larger than in the Low baseline – High change and the High baseline – High change groups (pairwise Wilcoxon rank sum tests adjusted for multiple comparisons p-value < 0.001).

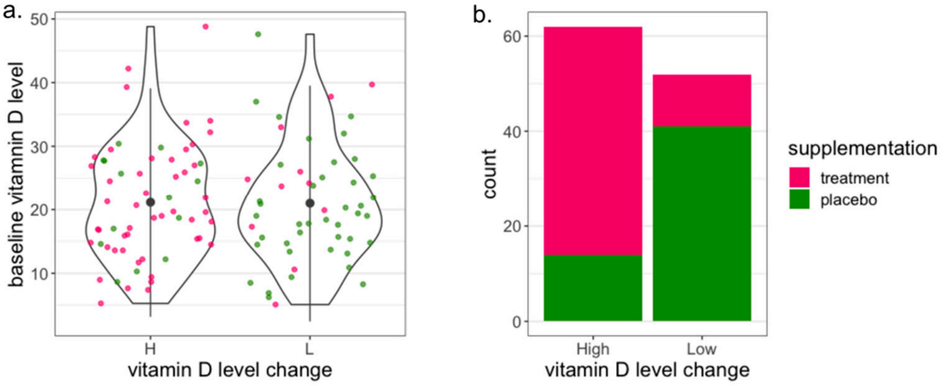

Figure S3 a. Distribution of the participants change in vitamin D level over the trial period with respect to their baseline vitamin D; they are not significantly associated. **b.** Treatment assignement of the participants in both levels of vitamin D level change. There is a significant association between the two (Chi squared test p-value < 6.6×10^{-9}).

Supplemental tables

			baseline - change				
		**Overall**	**High - High**	**High - Low**	**Low - High**	**Low - How**	***p* value**
		114	29	22	33	30	
**Race (%)**	Black	53 (46.5)	5 (17.2)	8 (36.4)	23 (69.7)	17 (56.7)	< 0.001
	Other	34 (29.8)	11 (37.9)	5 (22.7)	9 (27.3)	9 (30.0)	
	White	27 (23.7)	13 (44.8)	9 (40.9)	1 (3.0)	4 (13.3)	
**Household income (%)**	50k or more	35 (30.7)	15 (51.7)	9 (40.9)	4 (12.1)	7 (23.3)	0.021
	Less than 50k	50 (43.9)	8 (27.6)	10 (45.5)	19 (57.6)	13 (43.3)	
	NA	29 (25.4)	6 (20.7)	3 (13.6)	10 (30.3)	10 (33.3)	
**Education (%)**	College or more	94 (82.5)	29 (100.0)	17 (77.3)	23 (69.7)	25 (83.3)	0.016
	Highschool, technical school or less	20 (17.5)	0 (0.0)	5 (22.7)	10 (30.3)	5 (16.7)	
**Baseline vitamin D level (mean (SD))**		21.08 (9.07)	28.96 (6.09)	29.57 (6.76)	14.28 (4.14)	14.71 (4.59)	< 0.001
**Site name (%)**	Boston	51 (44.7)	10 (34.5)	11 (50.0)	18 (54.5)	12 (40.0)	0.005
	San Diego	15 (13.2)	10 (34.5)	1 (4.5)	0 (0.0)	4 (13.3)	
	Saint Louis	48 (42.1)	9 (31.0)	10 (45.5)	15 (45.5)	14 (46.7)	
**Maternal asthma (%)**	No	75 (65.8)	20 (69.0)	16 (72.7)	22 (66.7)	17 (56.7)	0.632
	Yes	39 (34.2)	9 (31.0)	6 (27.3)	11 (33.3)	13 (43.3)	
**Maternal hay fever (%)**	No	49 (43.0)	9 (31.0)	13 (59.1)	17 (51.5)	10 (33.3)	0.105
	Yes	65 (57.0)	20 (69.0)	9 (40.9)	16 (48.5)	20 (66.7)	
**Maternal age (mean (SD))**		27.56 (5.85)	29.06 (5.13)	27.93 (6.08)	25.85 (5.90)	27.73 (6.07)	0.184

Table S1 Summary of the participants' characteristics with respect to their baseline and change in vitamin D level. Participants with a baseline vitamin D level above (below) the mean (21.08 ng/mL) were placed in a *High (Low) baseline* group. Participants with a change higher than 10 ng/mL were placed in a *High change* group, and those with a maximum change of 10 ng/mL were placed in a *Low change* group. In total there are four baseline – change groups: High (baseline) – High (change), High – Low, Low – High, and Low – Low.

			Vitamin D level change		
		**Overall**<br>114	**High (>10ng/mL)**<br>62	**Low (<=10ng/mL)**<br>52	***p* value**
**Race (%)**	Black	53 (46.5)	28 (45.2)	25 (48.1)	0.822
	Other	34 (29.8)	20 (32.3)	14 (26.9)	
	White	27 (23.7)	14 (22.6)	13 (25.0)	
**Household income (%)**	50k or more	35 (30.7)	19 (30.6)	16 (30.8)	0.995
	Less than 50k	50 (43.9)	27 (43.5)	23 (44.2)	
	NA	29 (25.4)	16 (25.8)	13 (25.0)	
**Education (%)**	College or more	94 (82.5)	52 (83.9)	42 (80.8)	0.852
	Highschool, technical school or less	20 (17.5)	10 (16.1)	10 (19.2)	
**Baseline vitamin D level (mean (SD))**		21.08 (9.07)	21.15 (8.98)	21.00 (9.26)	0.929
**Site name (%)**	Boston	51 (44.7)	28 (45.2)	23 (44.2)	0.525
	San Diego	15 (13.2)	10 (16.1)	5 (9.6)	
	Saint Louis	48 (42.1)	24 (38.7)	24 (46.2)	
**Maternal asthma (%)**	No	75 (65.8)	42 (67.7)	33 (63.5)	0.778
	Yes	39 (34.2)	20 (32.3)	19 (36.5)	
**Maternal hay fever (%)**	No	49 (43.0)	26 (41.9)	23 (44.2)	0.955
	Yes	65 (57.0)	36 (58.1)	29 (55.8)	
**Maternal age (mean (SD))**		27.56 (5.85)	27.35 (5.74)	27.81 (6.01)	0.675

Table S2 Summary of the participants' characteristics with respect to the change in their vitamin D level. The difference in vitamin D level was calculated for every subject as their final vitamin D level minus their baseline vitamin D level. Participants with a change higher than 10 ng/mL were placed in the High change group, and those with a maximum change of 10 ng/mL were placed in the low change group.
